# A case of unilateral keloid after bilateral breast reduction

**DOI:** 10.1186/1477-7800-5-3

**Published:** 2008-02-24

**Authors:** Haresh Devalia, Lucy Mansfield, Neda Minakaran, Dibyesh Banerjee

**Affiliations:** 1 St George's Hospital, Blackshaw Road, London, SW17 0QT, UK

## Abstract

Keloid scar is a manifestation of abnormal wound healing in predisposed individuals. Many treatment modalities have been tried with varying degrees of success. Radiotherapy is one such modality that is widely recognised. We present a case report and literature review based on a patient who developed unilateral keloid scarring following bilateral breast reduction surgery. Some 4 years previously, she had undergone breast conserving surgery followed by adjuvant radiotherapy for breast cancer. After her breast reduction surgery, she developed keloid scarring on the non-irradiated breast only. This case highlights a possible 'preventative' effect of radiotherapy in keloid formation.

## Background

Keloid scars can be an unsightly complication of surgery in predisposed individuals. Radiotherapy is a treatment modality that is widely recognised. We present a patient who received adjuvant radiotherapy to one breast for the management of breast cancer. She then underwent bilateral breast reduction surgery 4 years later and developed keloid scarring in the non-irradiated breast only.

## Case report

A 35-year old West Indian female was diagnosed with carcinoma in her left breast. She was treated by breast conserving surgery in 2001, followed by radiotherapy (50 Gy) to the left breast. Histopathology confirmed a 21 mm, grade 2, mucinous adenocarcinoma with no axillary lymph node involvement.

In 2005, she underwent bilateral breast reduction for relief of symptoms of macromastia. A few weeks post-operatively the peri-areolar scar on the right breast started becoming hypertrophic. Over the next few months it continued to grow in all directions, despite topical silicone gel treatment. Interestingly, the scar on the left breast healed without any complications, most probably due to the protective effect of prior radiotherapy. This case report demonstrates the effect that radiotherapy had in preventing keloid formation.

## Discussion

Keloid scars are abnormal wound responses in predisposed individuals. These fibrous growths result from a connective tissue response to trauma, inflammation, surgery, or burns. Classically they are described as healed human skin that extends beyond the confines of the original wound and are characterized by over abundant collagen deposition [1]. Keloids occur in all races, with preponderance in Africans [2]. Genetic predisposition appears to be a major factor in the role of keloid development (coupled with some form of skin trauma); however the mode of inheritance remains to be elucidated [3]. Regional susceptibility to keloid scarring is also recognized, with the presternal area, the back and the posterior neck being the most common sites [4].

Skin or wound tension has also been implicated as a critical factor in the development of keloids [5]. Several sources have been identified to be a cause of this tension. With loss of tissue (such as with the surgical excision demonstrated in this case) there will be an increased tension when an attempt is made to close the wound. In addition, underlying bony and cartilaginous skeleton will transmit a constant tension to the overlying skin.

Keloid therapy is fraught with varying degrees of success. The variety of treatments elucidates to the fact that few are satisfactory. Surgery alone has been documented to lead to recurrence rates of 45 to 100% [6]. Treatments that have been tried with limited success over the years include cryosurgery, laser destruction, hyaluronidase, nitrogen mustard, methotrexate, steroid infiltration, retinoic acid, zinc, colchine, anthistamines, tetrahyroxyquinone, compression splints and dermabrasion. Many anecdotal reports of therapeutic success have been demonstrated to be untrue when investigated within the setting of randomized clinical trials.

Radiotherapy was first introduced as a treatment for keloid scars in 1906 [7]. Keloids remain the commonest type of benign disease treated by radiotherapy [8]. This can be attributed to the fact that these scars are particularly refractory to most other treatment modalities. Radiation can be used as a monotherapy or combined with surgery to prevent recurrence of keloids following excision. A combination of the two appears to be a superior approach to the management. When used as a monotherapy, recurrence rates of between 50 and 100% have been reported [9], unless large doses are used. A retrospective analysis with up to 5-year outcome data by Ragoowansi et al [10] revealed that the majority of patients' keloids can be controlled by a single operation with immediate adjuvant single fraction radiotherapy. From a review of other previous studies, reported recurrence rates at one year or more vary from 53% [11] to 2% [9]. It is difficult to make meaningful comparisons between these studies as there was such variation between treatment schedules. Within the literature there is no consensus as to the optimal dosage, fractionation or timing with respect to surgical procedures. Radiotherapy doses have been given over the range of 2 to 62 Gy [12].

The risk of radiation-induced malignancy may result in hesitation amongst doctors to recommend radiotherapy for treatment of such a "benign" condition. It has been shown in epidemiological studies that ionizing radiation carries a risk of carcinogenesis [8]. In particular, an increased incidence of breast cancer has been observed in women given radiotherapy for post-partum mastitis [13]. Despite the potential risks, however, there are very few reports in the literature of malignancies arising from the treatment of keloids with radiotherapy. The few that are reported are in patients irradiated at a young age, which appears to be the greatest risk factor [14,15]. The total-body radiation dose from a superficial low-voltage radiotherapy technique is low, thus the main excess risk would be anticipated to occur close to the treated site. As the risks remain yet to be truly quantified, the physician must clearly clarify this to the patient during the consent procedure.

## Conclusion

This case report demonstrates the effect radiotherapy had in preventing keloid formation. Direct comparison can be made between both irradiated and non-irradiated skin within the same individual, thus eliminating many other variables. Not only did the patient not develop keloid scarring after her first operation to the breast, which was treated by radiotherapy post-surgery, but that same breast was also protected from keloid scarring 4 years later after the bilateral breast reduction surgery.

## Consent

Written informed consent was obtained from the patient for publication of this case report and any accompanying images. A copy of the written consent is available for review by the Editor-in-Chief of this journal.

## Competing interests

The author(s) declare that they have no competing interests.

## Authors' contributions

HD: Concept, design, literature search, manuscript preparation. LM: Literature search, manuscript preparation. NM: Literature search, manuscript preparation. DB: Concept, manuscript editing, manuscript review.

**Figure 1:**
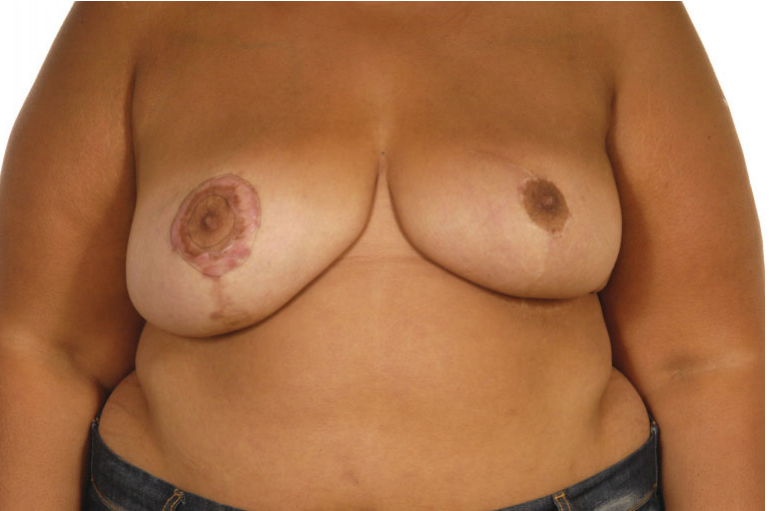
Photograph showing keloid scarring of right non-irradiated breast and absence of keloid scarring of left irradiated breast.

**Figure 2:**
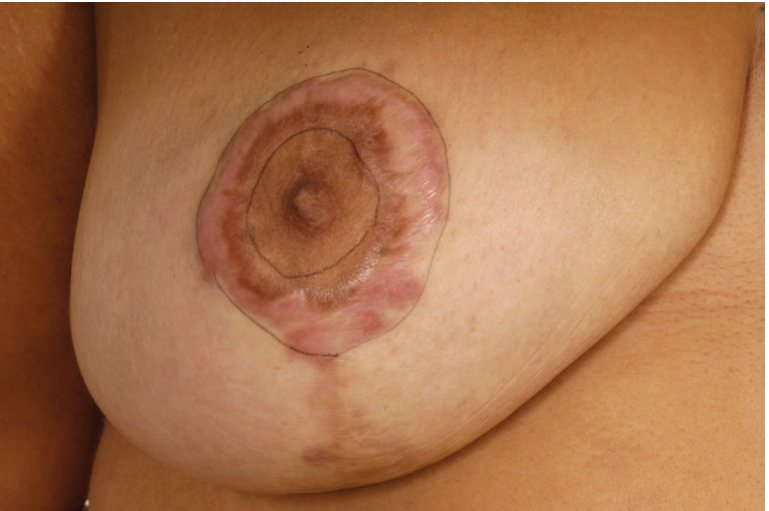
Photograph of right breast keloid scarring following bilateral breast reduction.

## References

[B1] Peacock EE, Madden JW, Trier WC (1970). Biologic basis for the treatment of keloids and hypertrophic scars. South Med J.

[B2] Oluwasanmi JO (1974). Keloids in the African. Clin Plast Surg.

[B3] Omo-Dare P (1975). Genetic studies on keloid. J Natl Med Assoc.

[B4] Kelly AP (1988). Keloids. Dermatol Clin.

[B5] Stegman SJ, Tromovitch TA, Glogan RG, Stegman SJ (1990). Treatment of keloids. Cosmetic Dermatologic Surgery.

[B6] Berman B, Bieley HC (1996). Adjuvant therapies to surgical management of keloids. Dermatol Surg.

[B7] De Bearman R, Gourgerot H (1906). Cheloides des maqueuses. Ann Dermatol Syphilol (Paris).

[B8] Botwood N, Lewanski, Lowdell C (1999). The risks of treating keloids with radiotherapy. Br J Radiol.

[B9] Borok TL, Bray M, Sinclair I (1988). Role of ionizing irradiation for 393 keloids. Int J Radiat Oncol Biol Phys.

[B10] Ragoowansi R, Cornes PG, Moss AL, Glees JP (2003). Treatment of keloids by surgical excision and immediate post-operative single-fraction radiotherapy. Plast Reconstr Surg.

[B11] Norris JE (1995). Superficial x-ray therapy in keloid management: A retrospective study of 24 cases and literature review. Plast Reconstr Surg.

[B12] Berman B, Bieley HC (1995). Keloids. J Am Acad Dermatol.

[B13] Mettler FA, Hemplemann LH, Dutton AM, Pifer JW, Toyooka ET, Ames WR (1969). Breast neoplasms in women treated with x-rays for acute post-partum mastitis; a pilot study. J Natl Cancer Inst.

[B14] Metayer C, Lynch CF, Clarke EA, Glimelius  B, Storm  H, Pukkala  E, Joensuu  T, van Leeuwen  FE, van't Veer  MB, Curtis  RE, Holowaty  EJ, Andersson  M, Wiklund  T, Gospodarowicz  M, Travis  LB (2000). Second cancers among long-term survivors of Hodgkin's disease diagnosed in childhood and adolescence. J Clin Oncol.

[B15] Yasui W, Tahara E (1999). Pediatric tumors and secondary cancer: The ninth International symposium of the Hiroshima cancer seminar, September Hiroshima, Japan. J Cancer Res Clin Oncol.

